# The everyday political economy of health: community health workers and the response to the 2015 Zika outbreak in Brazil

**DOI:** 10.1080/09692290.2019.1625800

**Published:** 2019-06-27

**Authors:** João Nunes

**Affiliations:** Department of Politics, University of York, Heslington, York, United Kingdom of Great Britain and Northern Ireland

**Keywords:** Everyday political economy, global health, community health workers, Zika, Brazil, neoliberalism

## Abstract

How is neoliberalism implicated in concrete health vulnerabilities? How do macro-level political economy, policy and institutions translate into everyday experiences? Drawing on Marxist, feminist and International Political Economy critiques of everyday life, the article advances an everyday political economy of health focused on four key components: power, agency, intersectionality and the mutual implication of the global and the local. These components enable a nuanced investigation of concrete experiences of health and disease, and of the local implementation of health policies in the context of neoliberalism. The framework is applied to the case of the 2015 public health response to Zika in Brazil, and specifically to the role of community health workers, close-to-community healthcare providers tasked with bridging the health system and vulnerable groups. The everyday practice of these workers, and their working conditions overwhelmingly characterized by precarity and low pay, reveal the presence of global neoliberal dynamics pertaining to the reconfiguration of the Brazilian state as healthcare provider in a context of encroaching austerity, privatization and narrowly-defined cost-efficiency. These dynamics impacted detrimentally upon the effectiveness of the Zika response.

In 2015, Brazil declared a war on mosquitoes. An outbreak of Zika virus disease, beginning in April in the northeast of the country, alarmed clinicians, researchers, policymakers, media and the public. Although Zika is a mild illness, causing fever, joint pain and headache, scientists puzzled over unprecedented symptoms in the most affected areas (Diniz, 2017). In November 2015, the Brazilian government declared a public health emergency based on the (then uncertain) association between Zika and neurological disorders. In February 2016, the World Health Organization declared Public Health Emergency of International Concern with a similar rationale (Heymann et al., 2016). Researchers later confirmed the link between Zika infection during pregnancy and a spectrum of disorders in newborns now termed Congenital Zika Syndrome (CZS), which ranges from learning difficulties to microcephaly (smaller head circumference) (Calvet et al., 2016). Zika was also associated with Guillain-Barré syndrome in adults, which leads to muscle weakness and paralysis, and can be life-threatening (dos Santos et al., 2016).

Because Zika is transmitted by mosquitoes, from the start the response focused on controlling the mosquito vector (Ventura, 2016). Health workers, authorities, the military and the population mobilized in a nationwide effort. The framing of Zika by the Brazilian government as fundamentally about mosquitoes obscured broader problems. The epidemic is part of a long-standing difficulty of the public health system in dealing with mosquito-borne diseases like dengue, chikungunya and yellow fever. In 2016 Brazil reported around 1.5 million cases of dengue and 609 related deaths. In the same year, 211,770 probable cases of Zika were reported (Secretaria de Vigilância em Saúde – Ministério da Saúde, 2016). Importantly, the narrow framing around the vector neglected the determinants of disease. The response overwhelmingly failed to address inadequate sanitation and urban planning, an under-resourced health system, economic inequalities and insufficient reproductive rights for women. Zika is a disease of poverty because mosquitoes reproduce where there is no reliable access to water, where families use tanks and makeshift receptacles and where rainwater and sewage run in the open air. Mosquitoes spread havoc when health systems cannot reach deprived areas and when the efforts of health workers are stifled by lack of resources, precarious working conditions, poor coordination and corruption (Lesser & Kitron, 2016). Thus Zika, like many other diseases, has a complex political economy, which is crucial to fully grasping exposure to disease. The poorest communities in Brazil were inordinately exposed due to insalubrious conditions and difficulties in accessing means of prevention like mosquito repellent and accurate information. Poverty also impacted upon the capacity to respond to Zika’s repercussions. For example, affordable, high-quality healthcare, namely specialized support like early stimulation, physiotherapy or speech therapy for children with CZS was difficult to access (Moreira, Mendes & Nascimento, 2018). Many mothers of children with CZS either lost or had to abandon their jobs because of care responsibilities and did not receive adequate support from the government and health authorities.

Zika speaks to academic works connecting health with socioeconomic factors like income, redistribution policies and public investment (Wilkinson & Pickett, 2010). In recent decades, with the growing importance of neoliberal globalization in global health policies (Keshavjee, 2014), scholars have considered the health implications of neoliberalism (Coburn, 2004; Navarro, 2007; De Vogli, 2011; Labonté & Stuckler, 2016). Sophie Harman (2011, p. 5) defines neoliberalism as ‘a global economic process that encompasses significant political and social reform based on the primacy of the market, competition, minimal state intervention, and private sector efficiency’. Neoliberalism, in the form of structural adjustment programmes and, more recently, austerity measures, has led to reduced public health investments in developed and developing countries, with detrimental impact upon public health systems (Rowden, 2013). Neoliberalism has resulted in the precarization of work, leading to economic insecurity and anxiety (Kalleberg, 2009). Schrecker and Bambra (2015) argue that neoliberalism has meant increasing material deprivation and economic inequality, along with the disappearance of social safety nets. Among its health impacts, they (2015, pp. 23–41) highlight food systems characterized by the production and consumption of cheap and harmful foods. Neoliberalism leads to obesity and malnutrition because many people cannot afford a healthy diet.

In sum, the literature associates neoliberalism with a pattern of adverse health effects. But how does neoliberal political economy produce these outcomes? The health literature often discusses neoliberalism in its easily recognizable characteristics, such as ideas and policies, without sufficient attention being given to how health effects are experienced, embodied and reproduced through informal and day-to-day practices. Neoliberalism becomes a short-hand for explaining harms in health that end up being treated as self-evident. This results in an impoverished understanding of how ill health is shaped by political economy. We need to go beyond the macro-level analysis of neoliberal arrangements and institutions, and focus on their manifestation at the micro-levels of communities, households and individuals. A concern with the micro-level is found in the work of Paul Farmer (2003), who draws on the concept of structural violence to explore the relationship between socioeconomic conditions and the perpetuation of suffering, and João Biehl (2007), who studies personal trajectories of AIDS patients against the background of governance structures.

How, then, is neoliberalism implicated in concrete health vulnerabilities? How do macro-level political economy, policy and institutions translate into everyday experiences? Speaking to this analytical challenge, this article develops an everyday political economy of health (EPEH) to explore the reproduction of ill health in the context of neoliberalism. The framework, drawing on the Marxist and feminist critiques of everyday life and on international political economy (IPE), is applied to the Brazilian response to the 2015 Zika outbreak. Zika is a remarkable case for examining the EPEH because it is a set of interlocking crises in everyday life. It draws attention to bodily matters that are often neglected, such as pregnancy, reproductive rights, disability and the unequal burden of care placed on women. The attention given to Zika as a health emergency obfuscated socioeconomic determinants and the ‘everyday emergencies’ experienced by millions of Brazilians.

Community health workers (CHWs) are here an entry-point for analyzing how the response to Zika materialized on the ground. CHWs are close-to-community healthcare providers (sometimes living in the communities they serve) who connect doctors and nurses with remote or hard-to-reach groups in many countries (Brownstein, Hirsch, Rosenthal, & Rush, 2011; Schneider, Lehmann, Schneider, & Lehmann, 2016). Despite having no specialized medical training, they play a critical role in providing access to health. They are therefore a unique lens for observing the social, political and economic dynamics shaping the Brazilian health system and the implementation of policies therein, and how these system-level drivers play out on everyday experiences. This study draws on fieldwork with 357 CHWs conducted between November 2016 and March 2017 in four municipalities in Minas Gerais, the second most populous (over 21 million) and fourth largest state in Brazil: Simonésia (population 19,633), Buritizeiro (28,335), Porteirinha (38,741) and Sete Lagoas (236,228) (Instituto Brasileiro de Geografia e Estatística, 2017). Fieldwork included semi-structured interviews with CHWs and their managers, focus groups with CHWs and shadowing of CHW household visits. It enabled an analysis of the concrete practices of CHWs during the Zika response, against the background of global neoliberal structures.

## The critique of everyday life

Ill health is not merely a bodily condition but a political and relational phenomenon (Venkatapuram, 2011, pp. 1–12). Social relations are organized so that some groups are systematically exposed to health risks – for example, by being unable to escape insalubrious conditions or poor diets, or by being unaware of hazards. Social relations also determine whether and how people are able to bounce back from disease. The relational dimension highlights the importance of the day-to-day practices through which some are rendered vulnerable, often by the actions of others with greater capabilities and resources. These micro-level practices need to be situated within a broader political and economic context to enable a more nuanced picture of how health and disease are produced in social systems.

The concept of the ‘everyday’ links the local with the global, in health and in other areas of life. This concept draws from the materialist philosophy of Marx and Engels, which holds that only the embodied, lived experience of humans – their working relations and interactions – can provide the basis for meaningful social analysis (Marx, 2000 [1932]; Engels, 2010 [1845]). In the wake of this insight, a critique of everyday life emerged, notably in the work of Henri Lefebvre (Lefebvre, 1991 [1958]; Lefebvre, 2002 [1961]). Lefebvre refused to discard everyday life as inconsequential. Rather, the everyday reveals the atomization of relations, commodification, bureaucratization, labor specialization, urbanization and separation of work from leisure that are intrinsic to capitalism (Gardiner, 2000, p. 13). For Lefebvre, the everyday is crucial to the reproduction of capitalism because alienation reaches into the most ordinary aspects of life. Alienation encompasses the economic, social, political and ideological spheres, shaping people’s opportunities, social networks, family relations, beliefs, habits and compulsions (Lefebvre, 1991 [1958], p. 167). Lefebvre argued that to understand everyday life we need to understand how society is organized, because the everyday is a level of social practice within broad structures and relations determined by capitalism (Lefebvre, 1991 [1958], p. 148). Simultaneously, the everyday is essential when coming to terms with capitalism. As Lefebvre (2002 [1961], 98, emphasis removed) put it, ‘[t]here can be no knowledge of society (as a whole) without critical knowledge of everyday life in its position… at the heart of this society and its history’.

While the Marxist critique of the everyday emphasizes the latter’s close connection with capitalism, feminist theory considers gender-based experiences to provide another critical engagement with the everyday. Highlighting the centrality of experience, Sandra Harding (1991, p. 310) described the feminist concern with difference, ambiguity and particularity as follows:

the subject/agent of feminist knowledge is multiple and sometimes even contradictory in that women are located in every class, race, sexuality, culture, and society. Feminist thought consequently is multiple and even contradictory as it starts from the lives of all these different kinds of women.

For feminists, lived experiences and daily interactions should be understood against the background of gender-based structures. These, in turn, relate to socioeconomic arrangements. The feminist and Marxist critiques of the everyday can therefore overlap. This can be seen in the work of Dorothy E. Smith (1987, p. 98), for whom a feminist approach seeks to grasp ‘an actual socially organized relation between the everyday world of experience and the social relations of capitalism’. Feminism adds value to the Marxist critique by considering the gendered dimension of the everyday in its connections with the ‘externalized and abstracted relations of economic processes and of the ruling apparatus in general’ (Smith, 1987, p. 99).

The critique of everyday life is also present in IPE works that consider how experiences are determined not only by domestic political and economic relations but also by global-level dynamics (Sinclair, 1999; Davies, 2006; Hobson & Seabrooke, 2008b). Everyday IPE argues that the everyday is essential for a nuanced understanding of the global, since the functioning of institutions – national, regional or transnational – depends upon ‘informal regimes that are created by everyday actors’ (Hobson & Seabrooke, 2008a, p. 9). Everyday IPE addresses a broad range of topics including: financial markets as embodied phenomena (Langley, 2008); the emergence of US financial power through practices and cultural norms (Konings, 2009); the reproduction of neoliberalism through precarious working relations (Cross, 2010); and international migratory flows of domestic workers (Elias, 2013). Everyday IPE also takes further the feminist insight that the personal is political and international. The gendered structures of everyday life are influenced and shaped by international actors (like transnational corporations) and forces (like structural adjustment programmes leading to privatization and precarization). At the same time, global-level arrangements depend upon the reproduction in everyday life of patriarchal relations that maintain gender inequality, overburdening women and placing them in vulnerable positions. For example, global economic processes rely on individual consumption patterns aligned with gender roles and expectations, as well as on the daily activities of traditionally neglected actors such as sweatshop workers, cleaners and sex workers (Elias & Roberts, 2016).

The Marxist, feminist and IPE approaches converge in seeing everyday life as more than the aggregate of routine activities pertaining to work, family life and leisure. They conceive the everyday as a lens for scrutinizing socioeconomic arrangements at the domestic and international levels. Their critique of the everyday sees neoliberalism as more than an abstract concept, paving the ground for an engagement with ‘actually existing’ (Wacquant, 2012) neoliberal structures and relations, and the ways they reproduce inequalities and vulnerabilities. The critique of everyday life is yet to be systematically applied to the study of health. This is an important gap since health is a site where the everyday ramifications of the political economy are particularly pronounced.

## The everyday political economy of health

The everyday political economy of health (EPEH) starts from the assumption that health policy design and implementation are the result of tensions, negotiations and struggles between actors – and are thus traversed by power relations. As Michael Gardiner (2000, 7, emphasis in the original) notes, the critique of the everyday zooms in on concrete activities to identify ‘the asymmetrical *power relations* that exist between a given bureaucratic or institutional systems and its users’. EPEH looks at the day-to-day implementation and embodiment of health policies, asking how power relations lead to the reproduction of health inequalities. Drawing on the Marxist and feminist critiques, as well as on IPE, it becomes possible to conceptualize power as a complex and multilayered phenomenon. Power in health is not something that simply emanates ‘top-down’ from the laws or coercive apparatus of the state, or from conditionalities and sanctions imposed by international actors. An everyday perspective allows for power to be scrutinized also in micro-level interactions. Didier Fassin (2012) observed that power in health circulates ‘horizontally’ through nodes in the public and private sector, as well as in civil society. In these interactions, power works through attempts to shape behavior in non-coercive ways. As Michel Foucault (1990 [1976], pp. 135–159) observed, power is not merely constraining since it promotes ways of living. Power emerges as a productive force ‘shaping and governing the capacities, competences and wills of subjects’ (Rose, 1996, 58). In addition to shaping behavior, power constitutes subjects by helping to determine their tastes, desires, self-understandings and dispositions to act – in short, their conduct. By seeping through the minutiae of everyday life, power directs the interests of actors towards economically useful directions. Subjects are not defined in opposition to power, but rather constituted by power relations alongside the reproduction of political and economic orders – indeed Foucault (2008 [2004], p. 271) wrote of the ‘homo oeconomicus of neo-liberalism’ as ‘a certain kind of subject who enabled an art of government to be determined according to the principle of economy’.

This constitutive dimension of power can be seen for example in health promotion initiatives that incentivize ways of acting and thinking not only for wellbeing but also to guarantee savings for the public health system. A contrasting example is advertising, through which unhealthy foods, sugary drinks or alcohol are presented as part of a desirable lifestyle, with profit motivations. By conjoining insights from Marxism and feminism, the everyday perspective is well-suited to recognize these attempts to shape the conduct of individuals and groups, from their assumptions and values to their activities (like consumption, exercise and hygiene). It directs the focus to the concrete ways in which power relations are embodied and translated in the diversity of daily experience – an important trope in feminist research. At the same time, in line with its Marxist credentials, EPEH frames the bodily effects of power as part of a broader set of socioeconomic arrangements.

The focus on the horizontal aspects of power should not obscure the fact that power relations are often underpinned by significant disparity. The shaping of behavior and the constitution of subjects are embedded within a context of unequal life conditions. Power relations occur in, and are themselves involved in the production of, an uneven environment in which certain groups are systematically placed in a position of subordination in relation to others (Young, 2011 [1990]). The inequality stemming from power relations does not necessarily result from a conscious and overt intention on the part of privileged groups (Hearn, 2008). Nonetheless, power circulates in an uneven playing field, one in which actors have unequal capacities and different degrees of control over the terms of the interaction.

EPEH conceives the horizontal approach of power, according to which the latter circulates between different nodes in society, as intertwined with these hierarchical effects. The Marxist lens is well placed to examine how a society traversed by unequal power relations is one where health vulnerabilities are unevenly distributed and where people can have markedly different capacities to respond to disease. For example, the weakening of public health systems by austerity policies and privatization helps to explain the adverse effects of seemingly horizontal relations underpinned by market-based logics, which in fact place disadvantaged sectors of the population in harm’s way. Moreover, by drawing on feminism, the everyday approach is able to adopt an intersectional perspective to study how power leads to interlocking inequalities. With its focus on everyday practice and bodily experience, EPEH supplements studies that have looked at how class intersects with gender (Moss, 2002; Iyer, Sen, & Östlin, 2008) and race (Kawachi, Daniels, & Robinson, 2005) in the reproduction of ill health.

The complex understanding of power afforded by EPEH signals great unevenness in the ability to shape health outcomes – in other words, different capacities to exercise agency. EPEH mobilizes a sophisticated notion of agency, according to which actors are indeed constrained by unequal power relations but are not mere targets or passive recipients. Everyday life is embodied and experienced in the context of constraining structures and relations, but this does not entail powerlessness. As Hobson and Seabrooke (2008a, p. 18) recognize, ‘by selecting new behavioral conventions that meet with their welfare-enhancing interests (not just economic, but also social well-being)’, actors can respond meaningfully to their surroundings and even help to bring about a change in norms and the socioeconomic context. By focusing on the possibilities of agency under situations of inequality, EPEH recognizes complexity in the production of health outcomes. These stem not just from the influence of powerful actors but also from the everyday acts of resistance of the less powerful. One example of this kind of resistance is provided in Anderson and Patterson’s (2017) study of the implementation of AIDS programmes in Malawi and Zambia, which shows how local actors are dependent on donor programmes and yet manage to resist and influence them. They adjust these programmes to the local context and appropriate them (at least partially) for their own interests.

Finally, IPE enables the EPEH perspective to trace the interactions between micro-level of experiences and macro-level dynamics shaping global health. On the one hand, EPEH considers the local effects of the global, that is, how global political and economic arrangements are experienced and embodied. It sheds light upon the concrete effects of neoliberalism: its influence on patterns of health and disease, and on determinants; its impact on the responsibilities and capacity of states, private actors and global institutions as healthcare providers or funders; its role in shaping the working conditions of the health workforce; and its bodily manifestations along class, gender and race lines. On the other hand, EPEH sees everyday practices as essential for the reproduction of neoliberal ideas. For instance, privatization of health services and the commodification of health are supported by the everyday perceptions and behaviors of health system users and health workers, who embody existing arrangements and thereby help to perpetuate them.

The argument has laid out an EPEH centered on four interlocking components: an understanding of power as the production of unequal life conditions and chances; a nuanced view of agency; the value of intersectional analysis to unpack the effects of power and the possibilities for agency; and the recognition of the mutual constitution of the local and the global. The remainder of the article shows the added-value of this approach, focusing on the Brazilian response to the 2015 Zika outbreak and the role of the community health workforce therein.

## Community health workers and the Brazilian response to the 2015 Zika outbreak

Brazilian CHWs (*agentes comunitários de saúde*) are part of the (public) Unified Health System (*Sistema Único de Saúde*, SUS). The origins of the CHW programme can be traced to 1987, when community workers were deployed in the state of Ceará to reduce child mortality and provide employment for women in a period of economic crisis heightened by severe drought (Lotta, 2015, p. 100). The programme was expanded and incorporated into the Family Health Strategy (*Estratégia Saúde da Família*, ESF), which from the early 1990s was rolled out within the SUS. The ESF supplemented curative medicine in hospitals with close-to-community prevention, health promotion and rehabilitation. Interventions were undertaken by teams comprising general practitioners, dentists, nurses, nursing assistants and CHWs (Cordoba & Jeronimo, 2013). The role of CHWs is to bridge the health system and its users, particularly the most marginalized and vulnerable groups. Their responsibilities include: health education and health promotion activities; keeping records of individuals and families in their area; making regular household visits to monitor the vaccination of children; scheduling specialist appointments; advising on the correct use of medication; and contributing to mosquito-control campaigns (Ministério da Saúde, 2012).

There are currently over 264 thousand CHWs in Brazil (Ministério da Saúde, 2018). According to regional-level sociodemographic studies, they are overwhelmingly women, with percentages above 75% and in some cases up to 95% (Lino, de Melo Lanzoni, de Albuquerque, & Schveitzer, 2012; Musse, Marques, Lopes, Monteiro, & Santos, 2015; Simas & Pinto, 2017). Although the law requires CHWs to have at least nine years of formal education, the same studies reveal that most (typically over 65%) have completed secondary-level education. CHWs can be hired through public procurement or outsourced through civil society organizations or trade unions, with different (formal and informal) selection processes. This leads to various contractual arrangements – permanent, temporary, verbal contracts, informal contracts, bursaries, among others (Morosini, Corbo, & Guimarães, 2007; Lima & Cockell, 2008). CHWs normally come from the communities where they work, to foster relations of trust and a friendlier service. Often, they are providers in, and users of, the same healthcare facilities. Proximity with the community means that they experience first-hand the health and socioeconomic vulnerabilities they are tasked with addressing. Their daily practice reflects the formal aspects of the health system, the informal environment of policy implementation and the wider context, thus enabling the interconnection between the local and the global to be scrutinized. CHWs are therefore uniquely placed for an EPEH analysis.

### Power relations in the response to Zika

The Zika public health emergency provided an opportunity for governmental power to make itself visible. Power was present in ‘top-down’ government interventions, for example coercive entry into lots and houses when owners could not be identified or located. The government’s strategy also placed great emphasis upon individual actions and omissions as crucial drivers for the spread of Zika. One of the key elements of the response was to alter people’s behaviors. Even as government authorities sought to reassert their power over the territory, efforts were combined with an approach that emphasized individual responsibility and the need for individuals to take health matters in their own hands. Paradoxically, the strategy sought to advance the reach of governmental power in areas where the state is often absent (e.g., urban communities or ‘favelas’ controlled by drug trafficking), and simultaneously epitomized the shift towards individual-centered healthcare – one of the mainstays of neoliberalism in health as will be discussed below.

CHWs, acting as ‘transmission-belts’ of federal, state and municipal authorities, were instrumental in the implementation of this strategy. They were essential in ensuring the capillary reach of state power, from government offices in Brasília to the most remote backyard. CHWs were heavily involved in mosquito control, identifying vacant buildings, lots or dumpsters where mosquitoes could reproduce and filling in checklists during household visits. These checklists were meant to ensure that homes and backyards did not have mosquito ‘hot-spots’. CHWs checked that empty bottles were turned upside down, that garbage bins and bags were securely shut, and that sand was poured in plant pot saucers to prevent water from accumulating (Ministério da Saúde, 2016a). Despite seeming mundane and routine, checklists were a crucial tool used by the Ministry of Health to influence individual behavior. As employees of the state and trusted members of their communities, CHWs are in a good position to shape how ordinary citizens think and behave (Bornstein & Stotz, 2008b). During the Zika response, they were able to navigate between the horizontal and vertical facets of power, implementing government directives while interacting with the population to promote certain ways of thinking and acting.

The everyday practice of CHWs during the Zika epidemic underscored their position as important nodes in the circulation of power at the micro-level of health policy implementation. The checklists enabled state authorities to enhance their reach in the territory through the collection of information, which in turn opened the way for the regular deployment of response teams. Checklists also functioned as templates of ‘good behavior’, through which the health authorities sought to constitute subjects. With the latter purpose in mind, CHWs disseminated information about Zika, its symptoms and effects, and about vector control. This was done door-to-door and through community mobilization in *mutirões*, collective interventions drawing on voluntary collaboration between people with common interests (such as co-workers or neighbors). During the Zika epidemic, *mutirões* included distribution of leaflets, cleaning of vacant lots, information sessions for the elderly or school children, among others.

Community mobilization activities signal a broad political function of CHWs, who are more than healthcare providers since they can also play an important role as community organizers (Silva & Dalmaso, 2002). As observed during fieldwork, CHWs used government-mandated tasks as opportunities not only to exert influence over behavior but also to position themselves as powerful in their communities. Indeed, for many health-system users CHWs are the only face of government power. Because of their role as gatekeepers to essential health services, CHWs have power in relation to those more marginalized than themselves. Gabriela Lotta (2015) has argued that CHWs function as ‘street-level bureaucrats’ with significant discretion in the day-to-day interpretation and implementation of policies. They can determine who has access to specialist appointments and who will remain on the waiting list, a basic yet very effective power resource in impoverished environments. In a context of insufficient resources and great informality in policy implementation, CHWs routinely make allocation decisions based not only on the seriousness of health problems, but also drawing on their own classifications and judgments about ‘deserving’ and ‘undeserving’ people. Lotta observes, for example, that drug users and people with mental health problems are often seen as less deserving of health system resources due to their non-compliance with treatments, and because their condition sometimes leads them to fail to adhere to the advice and recommendations of CHWs. In other words, decisions made by CHWs exclude a patient profile that should be a priority. In these cases, non-compliance and non-adherence are further evidences of vulnerability, which is aggravated when CHWs categorize these people as undeserving and shut down part of the state’s doors to them.

The analysis of the everyday practice of CHWs shows a complex picture of power relations reproducing an uneven playing field. Although CHWs have succeeded in reducing inequalities in some cases (Peres, 2007), through the use of discretionary power they can in other situations contribute to their entrenchment. As Lotta's work shows, the production of inequalities by CHWs occurs in a material sense because their actions and decisions determine the nature, quality and quantity of services to be made available to health system users. CHWs also impact upon inequalities in a symbolic way as they judge each situation and classify already vulnerable users, thus potentially contributing to their further stigmatization.

### The ambiguities of CHW agency: an intersectional analysis

The everyday mobilization of CHW power reveals ambiguities in the agency of these workers. This ambiguity was visible and rendered more acute during the Zika epidemic. On the one hand, CHWs were instrumental in the circulation of power. The situation of public health emergency emphasized the individual behavior change task that CHWs are particularly well positioned to deliver. It underscored the power CHWs already hold by virtue of their bridging role between the health system and its users, their privileged access to government’s resources and their influence in resource allocation

On the other hand, however, the ability of CHWs to influence outcomes was severely constrained. This powerlessness had to do with the limitations of the vector control strategy CHWs were helping to implement, and its insufficient recognition of socioeconomic and environmental determinants, which undermined long-term effectiveness (ABRASCO - Associação Brasileira de Saúde Coletiva, 2016). Powerlessness also relates to structural issues in the SUS, which grapples with chronic underfunding and is simultaneously centralized at the federal level and dependent upon state- and municipal-levels – and hence upon the political will and probity of local officers – for the disbursement of funds and programme delivery. This has resulted in lack of coordination and unevenness in the quality of healthcare provision across the territory.

Coordination problems impacted upon the everyday work of CHWs during the Zika response. Interviewees expressed confusion about their responsibilities in vector control, particularly regarding their relationship with community workers devoted to endemic diseases (*agentes de combate a endemias*). These latter workers are also in close contact with populations and cover the same territories as CHWs. However, they depend upon the Health Surveillance branch of the Ministry of Health, whereas CHWs are tasked with primary healthcare responsibilities. In the territories studied during fieldwork, confusion about roles impaired the effectiveness of the response and added to the frustration of CHWs and communities.

The intersectional lens of EPEH sheds light upon a range of factors impairing the agency and effectiveness of CHWs during the Zika epidemic. Inspired by feminist insights, intersectionality allows for a consideration of the concrete and bodily effects of socioeconomic conditions, gender, race and age; how these connect in the local context; and how they relate with global dynamics. Precarious socioeconomic background and working conditions are a permanent feature of the everyday life of CHWs and played a major role in the Zika response. Brazilian CHWs are tasked with tackling a broad range of health vulnerabilities while living vulnerable lives (Brand, Antunes & Fontana, 2010; Alonso, Béguin & Duarte, 2018). The profession of CHW is overwhelmingly marked by the precarity of contracts, a situation that is difficult to overcome because of the emphasis placed upon proximity with the community. The idea that CHWs should live in the communities where they work has been one of the obstacles preventing a widespread use of public procurement, where proximity cannot be a criterion of selection (Nogueira, Silva & Ramos, 2000). Fieldwork in Minas Gerais revealed a general concern with low salary and lack of job security and career prospects. This concern is confirmed by studies in other regions (Bornstein & Stotz, 2008b; Baralhas & Pereira, 2011; Costa et al., 2012). In the Sete Lagoas municipality, for example, 70% of CHWs (192) were on fixed-term contracts; in Buritizeiro, the figure was 98% (51). For interviewees, this points to insufficient recognition by authorities and other health professionals of the importance of CHWs. The view, shared by some health professionals, of CHWs as easily-replaceable is compounded by the fact that the job of CHW is often seen as a ‘stop-gap’ by the workers themselves (Rosa, Bonfanti & Carvalho, 2012; Lopes et al., 2012).

CHWs are also severely overworked (Wai & Carvalho, 2009, Costa et al., 2012). They face constant demands from community members (including friends and family members) outside regular working hours (Veiga Martines & Corrêa Chaves, 2007). Proximity to the community also leaves CHWs vulnerable to harassment and invasions of privacy (Andrade Jardim & Lancman, 2009). Interviewees reported that the anxiety, uncertainty and misinformation surrounding Zika increased pressures upon them, as health authorities sought to ensure that surveillance checklists were being completed in a timely manner. In addition to responding to demands on weekends and holidays, several interviewees had to perform tasks, like cleaning, that went beyond their job description. Moreover, many interviewees rely on social media and mobile phone apps to reach users during working hours, when many people cannot be present during household visits. While this can be helpful in the fulfilment of their commitments, it also makes it harder to separate working hours from leisure.

Overwork is one of the factors leading to burnout, stress and other psychological and physical problems among CHWs (Telles & Pimenta, 2009; Mascarenhas, Prado & Fernandes, 2013). This is aggravated by the uneven coverage of healthcare provision for CHWs, notably mental healthcare (Maia, Silva & Mendes, 2011). Like other community members, CHWs face difficulties in accessing services in a timely manner. As revealed during fieldwork, the coverage of specialist appointments and mental health support for CHWs was inadequate and in some cases non-existent.

Training of CHWs also played a significant role during the Zika epidemic. Although following federal guidelines, training about Zika and its repercussions was organized at municipal level and often depended upon the initiative of a health clinic manager or nurse. Fieldwork showed great discrepancies in coverage, resulting in significant misconceptions among interviewees about the symptoms of Zika, its distinction from other mosquito-borne diseases like dengue and chikungunya, the dangers faced by pregnant women and new-borns. Zika evidenced a problem in the SUS with CHW training, which is overwhelmingly fragmented, unsystematic, uneven across the country and often deployed when CHWs are already on the job (Morosini, 2010). Angélica Fonseca (2016, p. 328, author's translation) observes that Zika revealed a ‘reversion in the professional training of these workers’, with reliance on short courses focused on specific interventions in lieu of a solid preparation. According to Fonseca, the short duration and contingent nature of training ties with the precarious employment of many CHWs. Because they are not expected to stay on the job for a long time, there are scarce incentives for systematic and in-depth training.

Therefore, on top of strategic misdirection and workforce coordination issues, precarious working conditions of CHWs became a problem during the Zika outbreak by impacting upon the ability of these workers to contribute. For example, inspecting people’s homes and backyards for mosquito breeding sites relied upon long-term relations with community members because granting access to CHWs is not mandatory and often occurs only after trust has been established. Simultaneously, delivery of vector control initiatives often required CHWs to go beyond official responsibilities and regular working hours. Many interviewees resented the extra burden that affected their other tasks, some of which were arguably more pressing.

The government’s strategy of changing individual behavior also suffered as checklists became the be-all and end-all of what should have been a sustained disease prevention and health promotion intervention. Changing people’s behavior required dispelling myths, for instance about homemade mosquito repellents, and promoting the correct use of contraception (Ministério da Saúde, 2016b). The success of these initiatives also hinged upon good relations between CHWs and communities. As reported by interviewees, CHWs needed to be seen as authoritative sources of knowledge by the population; on the other, sufficient familiarity was required so that the subject of contraception could be approached.

The problem with CHW working conditions during the Zika epidemic intersected with gender and race, becoming an acute bodily question. Brazilian CHWs are overwhelmingly poor and vulnerable women, many from non-white backgrounds. In the municipalities studied, 98% of CHWs were women. In Buritizeiro, 51 out of 52 CHWs were women; 32 self-identified as *parda* (brown), 13 as black and only 5 as white. Moreover, many CHWs are single mothers dealing single-handedly with childcare and household duties. In Buritizeiro, 41 out of 52 had children; 26 were single, 3 were divorced, and only 21 were married; and 31 (60%) were the main/sole provider in the household. Many CHWs, upon which society already places inordinate expectations of care, perceived Zika-related tasks as yet another burden. The stress felt by CHWs because of precarious and difficult working conditions was heightened by the fact that many were women in fertile age, one of the risk groups during the Zika emergency. In Sete Lagoas, 67% of CHWs (183) were under 40 years old; in Buritizeiro, the figure was 83% (43). For some, widespread fears in society about neurological disorders (like microcephaly) affecting new-borns as a result of Zika infection made Zika-related work particularly sensitive.

The working conditions of CHWs reflect an understanding of women as carers that permeates Brazil’s highly patriarchal society, with female labor perceived as a natural extension of domestic work (Barbosa, Menezes, David, & Bornstein, 2012). Female CHWs, already living in position of vulnerability, are expected to deliver assistance to their communities while receiving meagre economic rewards and little job security. Their precarization is also correlated to the feminization of the workplace, in which flexibility and precarity are accompanied by increasing gender inequality. As Regina Barbosa and co-authors (2012, 762, author's translation) put it, ‘this work modality appropriates gender to promote a function of care that becomes quite efficient in the reproduction of a social system that… deepens social inequalities at all levels’.

In sum, the intersectional lens reveals the ambiguous position of Brazilian CHWs, simultaneously health workers in a position of power and vulnerable community members. The Zika emergency heightened this tension and accentuated the paradoxes in CHW agency.

### Brazilian CHWs: local, national, global

For the EPEH perspective, micro-level vulnerabilities of CHWs reveal problems in Brazil’s public health, which in turn connect with global dynamics. One of these is the neglect of socioeconomic determinants. The narrowing of Zika as a mosquito problem and a question of individual behavior glossed over the context in which individual decisions are made. For example, calls for the use of mosquito repellent clashed with the fact that the poorest cannot afford certified products or are not aware of their importance (Gonçalves, Tenório & da Silva Ferraz, 2018). The requirement that families (most often women) remove mosquito breeding sites from their homes was undermined by poor sanitation infrastructure in many areas, which rendered individual efforts futile.

Insights from Marxism and from IPE are particularly useful for understanding how the local and the global were intertwined during the response to Zika. Emphasis on individual responsibility for health is part of an ongoing reconfiguration of the Brazilian state as healthcare provider, which connects with a global neoliberal context of shrinking public service provision. Since its inception, the SUS has coexisted with pushes towards privatization and marketization, and has grappled with funding shortages (Ocké-Reis, 2012). In recent years, the domestic context has become increasingly inhospitable to public healthcare provision. Since coming into office in August 2016, the Michel Temer government followed a neoliberal-inspired austerity ideology based on the reduction of public spending – the utmost example being the Constitutional Amendment 95, of the 15th December 2016, which froze federal primary expenditure for 20 years with profound implications for public health funding (Vieira & de Sá, 2016). Attempts to downsize the SUS were supported by the dissemination of negative visions about its inefficiency, bad resource management and the need to cut funds. For example, in July 2017, Ricardo Barros, then Minister of Health, criticized the management team of the public Hospital of São Paulo for refusing to ‘cut unnecessary expenses’, and stated that ‘we will no longer pay for inefficiencies’ (Mengue, 2017). CHWs are particularly vulnerable targets when public health is discredited in this way. Their functions require long-term work and have effects that are difficult to measure and quantify, going against the grain of the neoliberal logic of ‘value for money’.

The discrediting of the SUS by some politicians enables private health plans to be presented as a panacea to ensure efficiency, sustainability and proper resource use. As Ligia Bahia (2018) has observed, the private sector in Brazil succeeded in imposing restrictions upon the use of public funds and in advancing the market as alternative healthcare provider. Between 1998 and 2013, the percentage of Brazilians with private health plans rose from 24.5 to 27.9% (in the United Kingdom, the percentage rose from 0.2% in 2000 to 3.4% in 2014). In 2013, private expenditure in health, excluding expenses with medicines, was 33.7% in Brazil (the weight of private plans in total health expenditure is typically below 5% in many European countries; Bahia, Scheffer, Poz, & Travassos, 2016; Malta et al., 2017). The privatization of health in Brazil reflects the growing influence of transnational capital, often involved in electoral campaign financing (Scheffer, 2015).

This privatization represents a ‘ruthless model of social segregation’ (Bahia, 2018, p. 12). The users of the SUS, and beneficiaries of the CHW programme, are overwhelmingly female, black or brown, with little formal education and low per capita household income (Fernandes, Bertoldi & Barros, 2009; Silva, Ribeiro, Barata, & Almeida, 2011). The Brazilian CHW programme has been accused of contributing to a chasm between technologically developed services for privileged groups and a ‘low-tech’ assistance to the poor (Favoreto & Camargo Jr., 2002; Bornstein & Stotz, 2008a). According to this interpretation, and while they provide essential services to groups who would otherwise be neglected, CHWs end up becoming a ‘band-aid’ that epitomizes the structural exclusion of the poor from the highest standards of healthcare.

The everyday practice of CHWs confirms this segregation. Whilst everyone can benefit from CHW visits, this does not necessarily mean access to the full range of services available in the SUS. Medium-complexity services such as specialist appointments are limited due to under-resourcing and have been identified as a ‘bottleneck’ (Spedo, Pinto & Tanaka, 2010). The number of appointments that CHWs can provide is well below actual needs, and poor people can wait months to access services that others will pay for in the private sector. As reported by interviewees, delays lead to resentment among the population and reinforce views of public healthcare as ineffective – thus giving further clout to arguments for privatization. The agency of CHWs is co-opted in the implementation and reproduction of a system that, in its current form, breeds inequality in everyday life and ultimately undermines the idea of health as a public good.

## Conclusion

The framing of the 2015 Zika epidemic in Brazil as a war between a nation and an insect obscured the everyday emergencies experienced by millions of Brazilians, for whom the daily reality is one of deprivation, vulnerability and harm, coupled with unequal access to quality healthcare. Zika was, for a while, a matter of high politics, but it also signaled the persistent neglect of disease determinants and deep-seated inequalities.

In this article, Zika was the starting-point for an analysis of the everyday manifestations and reproduction of neoliberalism in health. The initial hypothesis was that the everyday emergencies of Brazil’s neglected populations have more to do with economic structures than with mosquitoes. The argument addressed the following question: can the health vulnerabilities witnessed during the epidemic be conceived as concrete effects of macro-level neoliberal economy and policies? Zika is a good case for unpacking lived experiences of neoliberalism since it raises issues pertaining to poverty, sanitation, urban planning, but also gender relations, race, pregnancy, disability and contraception. To shed light upon these issues, the argument explored the role of CHWs, a traditionally neglected component of the health workforce. Situated in an ambiguous position as healthcare providers and vulnerable community members, CHWs provide a unique grassroots perspective on the public health response to Zika and on the Brazilian public health system.

To explore the concrete effects of neoliberalism the paper developed an EPEH with four components: power, agency, intersectionality and the mutual implication between the local and the global. The case of Brazilian CHWs in the Zika response demonstrated the analytical added-value of this perspective, and particularly the benefits of mobilizing Marxist, feminist and IPE lenses to scrutinize everyday life. The everyday perspective contributes to understanding how health policies are implemented, and the intersections of the global and the local therein. The everyday practice of CHWs during the Zika epidemic revealed the influence of neoliberalism and showed global-level dynamics being reproduced locally. Neoliberalism permeated everyday life through mundane but no less meaningful practices (such as filling in checklists to measure success) and the circulation of power (the distribution of rewards and punishments to ‘deserving’ and ‘undeserving’ users because of resource scarcity). What happened at the level of communities, individuals and bodies during the response to Zika can be traced back to global economic policies. Simultaneously, global dynamics can only be fully grasped when seen as lived, experienced and embodied in the everyday.

The case of Brazilian CHWs thus contributes to a Marxist-inspired interpretation of the contemporary trajectory of global health, illustrating the global neoliberal trend towards privatization, the erosion of public healthcare provision and the precarization of labor, particularly of female labor. The case also confirms the value of (feminist-inspired) intersectional analysis for global health, showing that the success of health policies needs to be assessed alongside a consideration of their bodily effects along gender, race and class lines. Marxism and feminism combined allow us to examine in depth the ways in which inequality may be reproduced through the very processes of implementing health interventions – and not simply by the absence of policy.

In addition, the Marxist and feminist lens are relevant in that they point towards possibilities for political change. The everyday perspective presented a complex picture of CHW power and agency as constrained but never fully determined. As an ‘army’ of precarious, easily disposable workers, CHWs can be considered the weakest link the public health system. Nonetheless, as a professional category they are instrumental to realizing the universalist mission of the Brazilian public health system, while meeting the demands of extremely diverse territories and populations. The everyday functioning of the system is reliant upon the agency of CHWs, who engages in informal, power-laden and gender-infused interactions in their everyday practice. EPEH reveals an underlying tension in public healthcare provision in Brazil, showing how it constrains the agency of vulnerable CHWs while remaining heavily dependent on their insider knowledge of communities and on their capacity to make pragmatic decisions (e.g., about the allocation of resources), and even to improvise when faced with unforeseen demands and obstacles.

This tension constitutes a fault line where ‘top down’ rules and directives can be reinterpreted, reconfigured, resisted and subverted in the everyday. The Brazil case shows that CHWs are embedded in and sometimes help to reproduce the very social and economic arrangements that contribute to their own vulnerabilities and those of their communities. They do so by reproducing inequality through their discretionary power, and by being co-opted in an increasingly segregated system where the poor remain unable to access the full range of health services available. At the same time, because of their unique position in the communities and in the health system, CHWs are well placed to unlock the latter’s transformative possibilities.

Emancipatory elements have always been present in the trajectory of Brazilian public health. These include the idea of health as a collective project (*saúde coletiva*), the importance of community-based responses and public participation, the focus on disease determinants, and the engagement with popular and indigenous forms of knowledge. CHWs can play a crucial role in turning these ideas into reality, and their practice can thus potentially challenge global economic structures and their everyday manifestations. Despite the inhospitable environment – which has become even more difficult with Brazil’s shift to the far right after the October 2018 elections – the work of CHWs reveals at times the everyday contestation of the neoliberal model. Everyday acts of resistance and subversion can provide the seeds for broader change. An image sticks to mind from one of the several *mutirões* organized by CHWs across Brazil during the Zika outbreak: a colorful street parade with festive music, a female CHW disguised as a mosquito, children being alerted to the need to protect their health and that of their loved ones and neighbors. A message of community mobilization and solidarity was transmitted. This happened in Jardim Nazareth, a small neighborhood in São Bernardo do Campo, a city of around 817 thousand people situated in the outskirts of São Paulo, a megalopolis of more than 13 million. The Jardim Nazareth CHWs ironically enacted and subverted the reality of a country at war. No fighting was involved: it was a celebration of life.
